# Understanding the Complexities of Student Learning Progress in Texas: A Study of COVID-19 and Rural vs. Non-Rural Districts

**DOI:** 10.3390/bs14050408

**Published:** 2024-05-14

**Authors:** Shifang Tang, Zhuoying Wang, Lei Zhang, David Jimenez

**Affiliations:** 1 Department of Psychology and Special Education, College of Education and Human Services, Texas A&M University—Commerce, Commerce, TX 75428, USA; lzhang7@leomail.tamuc.edu; 2 Texas Advanced Computing Center, The University of Texas at Austin, Austin, TX 78712, USA; zwang@tacc.utexas.edu; 3 School of Education and Human Development, Texas A&M University, Corpus Christi, TX 78406, USA; david.jimenez@tamucc.edu

**Keywords:** COVID-19, academic achievement, rural, non-rural

## Abstract

In this study, we investigate the impact of COVID-19 on academic achievement in Texas public schools. Demographic and Grade 5 STAAR test data were collected from 1155 public school districts for 2018–2019 and 2020–2021. Multiple regression was adopted to analyze the differences between rural and non-rural districts, as well as the impact of demographic characteristics on students’ achievement. The results reveal significant differences in demographic characteristics between the two academic years, with non-rural districts exhibiting a greater decline in academic achievement than rural districts. Additionally, the findings suggest that higher teacher salaries correlate with better academic performance across various subjects and that English learners require additional support to acquire content knowledge and skills. We further confirm that the COVID-19 pandemic has disrupted the academic learning experience of Texas students, with rural districts displaying more resilience than non-rural districts.

## 1. Introduction

Many governments chose to close schools for several weeks in the spring of 2020 due to the COVID-19 epidemic, among which the United States initiated a policy of lockdown to prevent and slow down the spread of the virus, and students started online schoolwork and lessons with the support of their teachers and parents [1]. However, the education system in the United States was unprepared for protracted closures. Although school closures were regarded as one of the most effective strategies for preventing the transmission of the virus [2], many educators and researchers are concerned about the impact of COVID-19-related school closures on student academic achievement and learning disparities. The detrimental impacts of physical school closures (e.g., summer vacation or natural catastrophe) on student academic performance are extensively established (e.g., [3,4]). Specifically, Hanushek and Woessmann [5] projected that COVID-19-related school closures had a negative impact on student attainment of 0.10 standard deviations. According to [6] systematic review, school closures during COVID-19 adversely affected student attainment, particularly among younger kids and those from low-socioeconomic status households. Ref. [7] anticipated that socioeconomic attainment inequalities would grow by up to 30%.

The shifting from in-person instruction to online or hybrid learning led to problems for educational institutions, teachers, parents, and students. To begin with, schools lacked the structure to provide effective and quality instruction to children after the shutdown [8]. Teachers were not fully prepared to face the challenges associated with online learning, including limited technical support [9,10,11], the heavy workload in course content preparation [9,11], difficulties to explain formulas and teach a subject related to numerical problems [10], and maintenance and supervision of the online classroom [9,10]. In the meantime, students had to overcome several obstacles in online learning, including limited access to the internet or laptops [9,11], lack of parental support and engagement with instructors [11,12], and mental health problems [9].

According to state data in Texas, in April 2020, 569 school districts declared closures due to coronavirus fears [13]. Although school districts were encouraged to continue educating all students, legislators from both political parties and school superintendents in Texas urged the state to cancel statewide testing out of concern that students would miss school days during an extended spring break [14]. In March 2020, the Texas governor waived the requirements for the annual academic assessment—State of Texas Assessment of Academic Readiness (STAAR)—for the school year 2020–2021. The Texas Education Agency (TEA) resumed the STAAR tests for all school systems and campuses in the school year 2021–2022. The preliminary STAAR data analysis concluded that COVID-19 contributes to learning loss and decreases academic performance measured by STAAR across grade levels [15]. Specifically, according to Texas Academic Performance Reports by TEA [16], 15% fewer students passed STAAR math, and 4% fewer passed STAAR reading.

Specifically for schools in rural areas, students’ learning loss and the possible factors that impacted their achievement might need a close look. For example, compared to non-rural school districts, rural school districts normally had significantly more students identified as economically disadvantaged [17], limited instructional expenditure [18], a higher student mobility rate [17,19], and a high teacher turnover rate [20,21]. Given the geographic isolation of rural schools, limited resources, and lack of support, rural schools face significant challenges in providing effective and quality professional development to teachers [22,23]. With the largest number of students enrolled in rural public schools [17], Texas not only faces challenges similar to other rural areas, such as low expenditure and professional isolation, but it also possesses some unique rural education characteristics [18]. For example, nationally, 3.5% of rural students were identified as English learners, while this figure in Texas is 8.2% [24]. It was found in previous studies that there exists an achievement gap among rural and non-rural school students in reading [17,18,25] and science [26]. While school location is often used as an indicator in educational research and policy making, what impacts students’ academic achievement is not the categorization of rural or non-rural, but the local demographic characteristics associated with the school districts [18].

In this study, we aimed to investigate the impact of COVID-19 on Texas school districts’ demographic characteristics and fifth-grade students’ learning progress in rural and non-rural areas. In the following section, we provide an overview of three key topics related to K–12 education, including the impact of COVID-19 on education, the influence of geographic and demographic factors on academic performance in rural school districts, and the demographic diversity of rural school districts in Texas.

## 2. The Impact of COVID-19 on K–12 Education: Challenges and Issues

The COVID-19 pandemic has greatly impacted student learning, teacher instruction, and school support for students and educators [27,28]. To ensure the continuity of student education, K–12 schools transitioned to virtual learning during the pandemic [29]. However, prolonged lockdowns, the requirement for extended virtual learning, and subsequent waves and mutations of COVID-19 have disrupted the traditional learning environment and are expected to persist in the upcoming school year [29,30]. The significant shift to online instruction has presented a multitude of challenges for teachers, students, and administrators [29,31]. Teachers had to adapt their instruction to suit the new learning environment, resulting in a roughly equal distribution of review and new content, with a smaller emphasis on review and a greater focus on new material [12,29]. Moreover, researchers have noted that teachers had to reduce instructional time during the sudden shift to online learning, resulting in a decline in reading and math scores for the semester starting in March 2020 [28].

Moreover, during the COVID-19 pandemic, school administrators have faced many challenges. The transition to remote teaching has required educators to adapt to new technological tools and platforms to support virtual learning, while also ensuring that all students have equal access to digital devices and internet connectivity [32]. Administrators have also been charged with developing and executing comprehensive plans to ensure that schools remain safe and healthy for students, teachers, and staff. These plans have involved considerable time and resources, including guaranteeing sufficient personal protective equipment, scheduling regular COVID-19 testing, and implementing contact tracing protocols [33,34]. Additionally, administrators have had to address students’ social and emotional needs, severely impacted by the pandemic, by providing counseling services and other support mechanisms [12,34]. Finally, the pandemic’s financial pressures have mounted, putting administrators in a difficult position to make challenging decisions on budget cuts and staffing levels while still providing quality education to students [32].

The emergence of the COVID-19 pandemic has also brought to the forefront the challenging circumstances encountered by low-income households and rural areas concerning internet connectivity [35]. Despite the initial expectation that remote learning would be a smooth and facile transition for students and their families equipped with multiple electronic devices and high-speed internet, the reality has been quite the opposite for those who lack a reliable internet connection [36,37]. The repercussions of this digital divide are extensive and significantly impact students’ access to education and educational outcomes [36,37]. Empirical evidence suggests that students who lack access to dependable internet connectivity are more prone to academic setbacks, leading to long-term adverse effects such as reduced lifetime earnings and limited opportunities [38]. Consequently, guaranteeing that every student has access to reliable internet and devices has become crucial in education [38,39,40]. Reflecting on the broader challenges of the COVID-19 pandemic, Anderson [41] highlights the significant stress placed on educators as they navigated the shift to emergency remote teaching (ERT), often under less-than-ideal circumstances. This situation illustrates the global struggle within the educational sector to maintain continuity in learning during unprecedented disruptions.

## 3. Geographic and Demographic Factors Affecting Academic Performance in Rural School Districts

The academic performance of students in rural school districts is influenced by many different factors, including both where they are located and the characteristics of the people living there. Regarding geography, rural school districts are uniquely affected by their environment and the communities they serve [42]. The economy of rural areas is typically reliant on sectors experiencing declining job opportunities, thereby constricting the availability of educational resources for students. Further, a lower population density can aggravate the dearth of resources in rural regions, impeding students’ academic growth [43].

Moreover, demographic elements play a crucial part in students’ academic progress. Disparities in socioeconomic status (SES) can considerably affect the achievement gap between students in rural and non-rural settings [44]. Moreover, English language proficiency is imperative to students’ academic success, specifically in reading, math, and science [18,45,46]. Recent research has also established a potent correlation between the teacher turnover rate and student mobility rate, along with their academic performance in diverse subjects [18,45,46].

The COVID-19 pandemic has highlighted the gap in internet access for low-income families and rural areas, especially in terms of remote learning [35]. Students who lack reliable internet and electronic devices face considerable educational disadvantages, potentially harming their future opportunities and earnings potential [38,40]. Reports by rural teachers suggest that remote learners are often the most underprivileged regarding technology access, resulting in less effective pedagogy [47]. While online learning has the potential to enhance learning outcomes for many students, thoughtful consideration is necessary to prevent worsening social and economic inequalities [48]. Families with lower incomes and those in rural areas encounter major obstacles to accessing steady internet and digital tools, limiting their chances to engage in online education [32]. A recent study by Bacher-Hicks et al. [36] discovered pronounced disparities in the utilization of online learning materials between regions with varying income levels, internet access, and school types. Families with low SES may also be restricted in terms of study space, electronic devices, internet access, and books, all of which can negatively impact their children’s online learning experience [49].

## 4. Demographic Diversity in Rural School Districts: A Case Study of Texas

Unlike community classification, district demographic characteristics account for a higher percentage of variance in students’ academic achievement [18,45,50]. Understanding the impact of demographic variables is crucial when examining the academic achievement of rural and non-rural district students. Rural school districts often face similar challenges in improving students’ academic performance due to their geographic location [51]. However, within rural areas, school districts exhibit significant diversity regarding demographic characteristics, resources, and student needs [52,53]. As a result, academic outcomes for students in rural communities vary significantly along demographic dimensions, including students’ SES and racial ethnicity, region, distance from urban communities, and local economies [50].

Texas is an illustrative case of rural school district diversity, with nearly 700,000 students enrolled in rural districts [17]. The state’s rural school districts exhibit significant demographic diversity, with Hispanic, African American, and Caucasian populations comprising the majority. Although Texas’s rural school districts face common challenges, such as low expenditure per student, inequitable funding, low transportation costs, high mobility rates, and high poverty rates [17], there is considerable variation within Texas’s rural districts. For example, Lindsay Independent School District (ISD) and Santa Maria ISD, identified as rural school districts, have exhibited vastly different demographic characteristics based on their annual Texas Academic Performance Reports (TAPR). In 2020–2021, 9.8% of students in Lindsay ISD were identified as economically disadvantaged (ED), and no students were identified as English learners (ELs). The district’s student mobility rate was 4.3%, and the teacher turnover rate was 9.6%. In addition, the average years of teaching experience was 15.1 years. In contrast, 98.5% of students in Santa Maria ISD were identified as ED, much higher than the average state level of 60.2%. Additionally, 38.6% of students were identified as ELs, and the district’s student mobility rate was 11.8%, with a teacher turnover rate of 10.8%. The average years of teacher experience was 8.2 years, less than the state average of 11.2 years. Therefore, it is important to consider the diversity of rural districts, the impact of demographic variables, the limitations of data sources, funding, and resource allocation, and the impact of geographic location to gain a comprehensive understanding of the unique challenges faced by these communities and how these factors impact academic outcomes.

## 5. Study Purpose and Research Questions

Previous studies indicated a need to revisit the possible differences between rural and non-rural school districts regarding students’ demographic characteristics and their academic gains before and after COVID-19. Two years after the pandemic, it is the right time to assess the impact of COVID-19 empirically on districts’ demographics and student academic gains, as well as how the changed demographics possibly impact student achievement at the district level. Therefore, in this study, we sought to address the following three research questions:

Research Question 1: What was the impact of COVID-19 on Texas school districts’ demographic characteristics, including instructional hours, principal experience, teacher experience, teacher and student ratio, teacher full-time equivalence, teacher salary, teacher turnover rate, student mobility rate, percentage of students identified as an English learner and percentage of students identified as economically challenged?

Research Question 2: What was the impact of district location (rural vs. non-rural) on Texas fifth-grade students’ learning progress (difference between 2019 and 2021) as measured by high-stakes reading, math, and science tests?

Research Question 3: What were the impacts of district location (rural vs. non-rural) and demographic characteristics on Texas fifth-grade students’ learning progress (difference between 2019 and 2021) as measured by high-stakes reading, math, and science tests?

## 6. Method

### 6.1. Research Design and Context

In accordance with the Texas Education Agency (TEA) guidelines outlined in 2020, a rural school district in Texas is defined as having an enrollment of either less than 300 students or an enrollment exceeding 300 students but less than the median district enrollment of the state, with an average enrollment growth rate of less than 20% over the past five years. In 2018–2019, TEA identified 466 rural school districts out of 1210 public school districts across the state of Texas.

To investigate the relationship between rural/non-rural status and academic achievement, we collected rural and non-rural district-level data about STAAR reading, math, and science through the Texas Assessment Management System (TAMS). More precisely, the data acquisition efforts targeted 5th-grade district-level data for both the 2018–2019 and 2020–2021 academic years. Our ultimate analytical sample consisted of 1155 public school districts, with 461 categorized as rural districts. District-level demographic data from the years 2018–2019 and 2020–2021 were gathered from the Texas Academic Performance Reports (TAPR), which included a range of essential indicators such as the percentage of instructional hours, principal and teacher experience, teacher–student ratio, average teacher salary, teacher turnover rate, student mobility rate, as well as the percentage of students identified as English learners and economically disadvantaged.

### 6.2. Measurement

STAAR is a standardized testing program aligned with the Texas Essential Knowledge and Skills (TEKS) curriculum standards. It evaluates students’ abilities in core subjects, including reading, math, science, and writing, from grades 3 to 8, and employs performance level descriptors with four rating levels. STAAR is a mandatory testing program administered by the state that aims to assess students’ competencies and skills in key subject areas, including reading and mathematics from grades 3 to 8, writing in grades 4 and 7, and science in grades 5 and 8. For eligible students whose primary language is Spanish, TEA provides the alternative STAAR Spanish to evaluate their math, reading, and science academic performance for grades 3–5. STAAR uses performance-level descriptors to capture students’ academic performance on both STAAR and STAAR Spanish assessments, utilizing four rating levels: Masters Grade Level, Meets Grade Level, Approaches Grade Level, and Did Not Meet Grade Level. This study focuses on the percentage of students who achieved the Approaches Grade Level in STAAR reading, math, and science tests. This rating level represents the basic level of academic proficiency and whether a student passes the test, including students rated as Approaches Grade Level, Meets Grade Level, and Masters Grade Level. According to the Texas Education Agency [54], “Approaches Grade Level” refers to students who demonstrate some ability to apply the knowledge and skills outlined by TEKS in a familiar context. Students classified at this performance level will likely make academic progress in the next grade with targeted academic intervention. Table 1 provides detailed information about the tests.

### 6.3. Data Analysis

RQ1 aimed to investigate whether there was a significant change in district-level demographic characteristics after the onset of the COVID-19 pandemic. To this end, we conducted ten paired *t*-tests, respectively, to examine potential differences before and after COVID-19 with respect to the teacher and student demographic characteristics at the district level. These demographic characteristics included the percentage of instructional hours, principal experience, teacher experience, teacher-to-student ratio, teacher full-time equivalence, teacher salary, teacher turnover rate, student mobility rate, percentage of students identified as ELs, and percentage of students identified as EC.

The aim of RQ2 was to assess whether there was a significant difference in students’ academic progress in reading, math, and science between rural and non-rural school districts after the COVID-19 pandemic. To address this research question, we conducted stepwise hierarchical multiple regression analyses, respectively, for three dependent variables. The dependent variables were students’ learning progress in the three subjects. Specifically, we calculated the students’ learning progress by subtracting the percentage of students who achieved Approaches Grade Level in the STAAR tests in 2019 from the percentage who achieved Approaches Grade Level in 2021. To address RQ2, we included the location of the school districts in Model 1 as the grouping variable.

Model 1:

Y = intercept + *b*_1_ × rural + error

where *b*_1_ is the coefficient of the school district as rural.

The aim of Research Question 3 was to investigate the additional impact of demographic characteristics on students’ learning progress. To address this research question, we conducted stepwise hierarchical multiple regression analyses, respectively, for three dependent variables. The dependent variables used were the same as in Research Question 2. We calculated the change in district-level demographic characteristics by subtracting the demographic characteristic values in 2019 from those in 2021 for each variable that showed a significant change in response to COVID-19. We repeated hierarchical multiple regression analyses three times to determine whether the changes in demographic characteristics could predict students’ learning progress in reading, math, and science above and beyond school district location, respectively. To address Research Question 3, the variables reflecting the differences in demographic characteristics, including the differences in principal experience, teacher experience, teacher-to-student ratio, teacher full-time equivalence, teacher salary, teacher turnover rate, student mobility rate, percentage of students identified as ELs, and percentage of students identified as EC, were added in Model 2, following the district location condition in Model 1.

Model 2:

Y = Intercept + *b*_1_ × rural + *b*_2_ × PrincipalExp + *b*_3_ × TeacherExp + *b*_4_ × T_Sratio + *b*_5_ × TeacherFull + *b*_6_ × TeacherSalary + *b*_7_ × Turnover + *b*_8_ × Mobility + *b*_9_ × EL + *b*_10_ × ED + Error (1)

where *b*_1_ is the coefficient of the district as rural, *b*_2_ is the coefficient of district-level principal experience, *b*_3_ is the coefficient of district-level teacher experience, *b*_4_ is the coefficient of the district-level teacher-to-student ratio, *b*_5_ is the coefficient of district-level teacher full-time equivalence, *b*_6_ is the coefficient of district-level teacher average salary, *b*_7_ is the coefficient of district-level teacher turnover rate, *b*_8_ is the coefficient of district-level mobility rate, *b*_9_ is the coefficient of district-level percentage of students identified as ED, and *b*_10_ is the coefficient of district-level percentage of students identified as ELs.

## 7. Results

In the results section, descriptive analyses were first conducted to present rural and non-rural school district students’ academic performance as measured by STAAR reading, math, and science tests in 2019 and 2021 (Table 2).

RQ1: What was the impact of COVID-19 on Texas school districts’ demographic characteristics, including instructional hours, principal experience, teacher experience, teacher-to-student ratio, teacher full-time equivalence, teacher salary, teacher turnover rate, student mobility rate, percentage of students identified as an English learner, and percentage of students identified as economically challenged?

The results of paired sample *t*-tests revealed that there was a statistically significant difference before and after COVID-19 in terms of principal experience (*p* = 0.030), teacher experience (*p* < 0.001), teacher-to-student ratio (*p* < 0.001), teacher full-time equivalence (*p* < 0.001), teacher average salary (*p* < 0.001), teacher turnover rate (*p* < 0.001), student mobility rate (*p* < 0.001), percentage of students identified as ELs (*p* < 0.001), and percentage of students identified as EC (*p* = 0.004). No significant difference was identified between the percentage of instructional hours before COVID-19 in 2019 and after COVID-19 in 2021 (*p* = 0.063; Table 3).

Research Question 2: What was the impact of district location (rural vs. non-rural) on Texas fifth-grade students’ learning progress (difference between 2019 and 2021) as measured by high-stakes reading, math, and science tests?

To answer the second research question, stepwise hierarchical multiple regression analyses were conducted, respectively, for three outcomes: reading, math and science. The results of Model 1 revealed that there was a statistically significant impact of district location on Texas fifth grade students’ learning progress as measured by G5 STAAR reading, math, and science tests. Specifically, non-rural school districts showed a larger learning loss in reading after COVID-19 compared to rural school districts by 2.41% of students who achieved Approaches Grade Level (*p* = 0.002) when other variables were controlled. In addition, non-rural school districts showed a larger learning loss in math after COVID-19 compared to rural school districts by 5.77% of students who achieved Approaches Grade Level (*p* < 0.001) when other variables were controlled. Finally, non-rural school districts showed a larger learning loss in science after COVID-19 compared to rural school districts by 4.82% of students who achieved Approaches Grade Level (*p* < 0.001) when other variables were controlled. See Table 4 for full details on Model 1.

Research Question 3: What were the impacts of district location (rural vs. non-rural) and demographic characteristics on Texas fifth-grade students’ learning progress (difference between 2019 and 2021) as measured by high-stakes reading, math, and science tests?

To address the third research question, hierarchical multiple regression analysis was conducted three times to determine if the change in demographic characteristics (principal experience, teacher experience, teacher-to-student ratio, teacher full-time equivalence, teacher salary, teacher turnover rate, student mobility rate, percentage of students identified as an English learner and percentage of students identified as economically challenged) improved the prediction of students’ academic progress indicated by the change in percentage of students achieving Approaches Grade Level in G5 STAAR tests over and above district location (rural vs. non-rural) in reading, math, and science, respectively. See Table 4 for full details on each regression model.

Adding the change in demographic variables to predict students’ learning progress in reading led to an increase in *R*^2^ of 0.013, Δ*F* (9,1126) = 1.692, *p* = 0.086. The full model of the change in demographic characteristics and rural location of a school district to predict fifth-grade students’ learning progress in reading (Model 2) was statistically significant, *R*^2^ = 0.022, *F*(10,1126) = 2.497, *p* = 0.006; adjusted *R*^2^ = 0.013. Non-rural school districts showed a larger learning loss in reading after COVID-19 compared to rural school districts by 2.04% (*p* = 0.012) when other variables were controlled. In addition, the change in teacher turnover rate and the change in average teacher salary significantly predicted students’ reading progress. Specifically, as the change in average teacher salary increased by one dollar, the expected students’ learning progress in reading increased by 0.0003% (*p* = 0.023), holding the other variables constant. As the change in teacher turnover rate increased by one percentage, the expected change in students’ learning progress in reading increased by 0.09% (*p* = 0.030), holding the other variables constant.

Adding the change in demographic variables to predict students’ math learning progress led to an *R*^2^ of 0.024, Δ*F* (9,1126) = 3.186, *p* < 0.001. The full model of the change in demographic characteristics and rural location of a school district to predict fifth-grade students’ learning progress in math (Model 2) was statistically significant, *R*^2^ = 0.055, *F*(10,1126) = 6.535, *p* < 0.001; adjusted *R*^2^ = 0.046. Non-rural school districts showed a larger learning loss in math after COVID-19 compared to rural school districts by 5.07% (*p* < 0.001) when other variables were controlled. In addition, the change in teacher experience, the change in the percentage of students identified as ELs, and the change in average teacher salary significantly predicted students’ math progress. Specifically, as the change in average teacher salary increased by one dollar, the expected students’ learning progress in math increased by 0.0005% (*p* = 0.005), holding the other variables constant. As the change in teacher experience increased by one year, the expected students’ learning progress in math decreased by 0.93% (*p* = 0.003), holding the other variables constant. As the change in the percentage of students identified as ELs increased by one percentage, the expected students’ learning progress in math decreased by 0.65% (*p* = 0.004), holding the other variables constant.

Adding the change in demographic variables to predict students’ learning progress in science led to an increase in *R*^2^ of 0.023, Δ*F* (9,1124) = 3.060, *p* = 0.001. The full model of the change in demographic characteristics and rural location of a school district to predict fifth-grade students’ learning progress in science (Model 2) was statistically significant, *R*^2^ = 0.044, *F*(10,1124) = 5.223, *p* < 0.001; adjusted *R*^2^ = 0.036. Non-rural school districts showed a larger learning loss in science after COVID-19 compared to rural school districts by 4.42% (*p* < 0.001) when other variables were controlled. In addition, the change in the teacher-to-student ratio, the change in the teacher average salary, the change in the percentage of students identified as ELs, and the change in student mobility rate significantly predicted students’ science progress. Specifically, as the teacher-to-student ratio change increased by one student per teacher, the expected students’ learning progress in science decreased by 1.225% (*p* = 0.004), holding other variables constant. As the change in average teacher salary increased by one dollar, the expected students’ learning progress in science increased by 0.0003% (*p* = 0.038), holding the other variables constant. As the change in student mobility rate increased by one percentage, the expected students’ learning progress in science increased by 0.32% (*p* = 0.020), holding the other variables constant. Moreover, as the change in the percentage of students identified as ELs increased by one percentage, the expected students’ learning progress in science decreased by 0.533% (*p* = 0.003), holding the other variables constant.

## 8. Discussion

### 8.1. Impact of COVID-19 on Demographic Characteristics

This study is a data-driven analysis to explore the impact of COVID-19 on Texas rural and non-rural school district 5th-grade students’ academic performance as measured by STAAR reading, math, and science tests. The overall findings indicated a significant difference in Texas school district demographic characteristics in 2019 and 2021. Among these variables, some changes are worth attention. Compared to 2019, there is a significant increase in teachers’ average salary in 2021. Moreover, student mobility and teacher turnover rates are significantly lower in 2021 compared to 2019. While students’ academic performance is often found to be associated with teacher turnover rate [55] and student mobility rate [56], the increase in these variables might not be strong enough to mitigate the impact of COVID-19 on students’ academic performance.

### 8.2. Impact of COVID-19 on Students’ Academic Performance

The hierarchical linear regression analysis results suggested that COVID-19 significantly negatively impacted students’ academic achievement across subjects, which is consistent with the report by TEA [16]. Both rural and non-rural districts declined in the percentage of students who achieved Approaches Grade Level on STAAR reading, math, and science tests after COVID-19. Specifically, COVID-19 had a more significant negative impact on non-rural districts than rural school districts. Before COVID-19, Texas rural school districts already faced the challenges of poverty [17], insufficient access to professional development [23], and racial diversity [17]. Researchers have been making efforts to support rural districts’ teachers and students. For example, it was found in a previous study that the virtual delivery of professional development and mentoring were effective and practical solutions for rural teachers to access quality pedagogical support to further improve students’ academic learning [57]. In light of the findings that non-rural districts experienced a greater learning loss compared to rural districts, a deeper analysis is warranted to understand the underlying factors contributing to this unexpected result. It is crucial to consider the unique characteristics and resources of rural districts in Texas, which may differ significantly from those in other regions or from the typical portrayals of rural education.

Firstly, the methodological design of our study, primarily relying on aggregated district-level data, may have influenced these findings. While this approach provides a broad overview, it potentially overlooks subtle intra-district variations that could explain better resilience in rural areas. Moreover, during the pandemic, Texas rural districts might have had distinct advantages that mitigated the impacts of school closures. For example, smaller school sizes and community cohesion typical of rural areas might have facilitated more effective communication and implementation of distance learning strategies. The role of local education authorities and their support during the pandemic, including the provisioning of technological resources and training, could have also played a crucial role in these districts. Additionally, our findings raise questions about the adequacy of the current definitions and classifications of ‘rural’ in educational research. The definition used in this study, as provided by the Texas Education Agency, might mask significant variability within rural districts—ranging from remote areas with severe resource limitations to those closer to urban centers that might not face the same challenges. This variability could inadvertently lead to findings that suggest a homogeneity within rural districts that does not exist. Reflecting on how rural areas are defined and ensuring these definitions accurately reflect the demographic and geographic realities could lead to more precise and actionable insights.

These unexpected findings emphasize the importance of contextual and demographic factors in assessing the impact of educational disruptions like those caused by the COVID-19 pandemic. Future studies can consider these elements to provide a more comprehensive understanding of the dynamics at play. This approach will not only enhance the accuracy of research outcomes but also contribute to the development of targeted educational policies and practices that can better support vulnerable populations during crises. In comparison, non-rural districts, which may lack sufficient equipment, technological support, and resources due to lower socioeconomic conditions, appear less prepared to handle the shifts in instructional delivery prompted by COVID-19.

In addition, the findings indicated a larger numerical decrease in math and science than in reading, which is also consistent with the report by TEA that there is a larger decline in math and science than reading at the state level [15]. A potential reason that might lead to the phenomenon is that effective math and science instruction is often embedded with hands-on experiments and in-person engagement. Texas school districts transitioned between in-person learning, virtual learning, and a hybrid mode from spring 2020 to fall 2021 due to COVID-19. However, educational institutions, teachers, and families are not fully prepared to adapt the curriculum and learning material to engage students during virtual learning. Another potential reason is that out of the classroom, students still have greater opportunities to apply their reading skills, such as reading with parents [58]. However, only some parents can or know how to help their kids practice math skills, especially at higher grade levels [58], or the skills and resources to conduct science experiments.

The findings of the study further indicated that adding a series of district-level demographic variables significantly improved the model prediction, which is consistent with Tang et al. 2021 [18] that district geographic location is not the key term, but the demographic characteristics associated with the district that showed significant impact on students’ academic performance. Specifically, we found one district-level demographic characteristic, teacher average salary, constantly and significantly impacted students’ academic performance across subjects (reading, math, and science). The finding is consistent with a previous study that higher teacher salary is associated with a decreased academic gap among students of diverse backgrounds [59]. In addition, the percentage of students identified as ELs showed a significant impact on STEM subjects (math and science), indicating that ELs need additional and quality support to acquire content knowledge and skills, as well as an academic language.

## 9. Conclusions and Limitations

COVID-19 has significantly impacted the education of students across the nation. According to estimates, as soon as COVID-19 struck, up to three million students in the United States withdrew their enrollment [60], with students from low socioeconomic position perhaps being the hardest hit [7,61]. Students who return to school will likely be further behind and have a wider range of academic abilities [4]. Like many other states across the nation, Texas had to shift to remote learning during the pandemic. This abrupt transition has disrupted students’ academic learning experiences across Texas. The results of the current study indicate that COVID-19 significantly negatively impacted both rural and non-rural fifth-grade students’ academic performance across subjects. More importantly, non-rural school districts exhibited larger learning losses than rural districts. This finding may seem unexpected given that rural areas typically have fewer resources than their non-rural counterparts. Several factors unique to the rural districts in Texas, such as smaller student-to-teacher ratios, strong community ties, or distinct administrative strategies, might have contributed to these unexpected outcomes.

The study has several limitations. First, our study only focuses on Texas fifth-grade students’ academic performance. However, the impact of the pandemic on students’ academic learning might not be the same at different grade levels or in different states. Future studies should consider investigating students’ learning loss across grade levels and in other states, or even across the nation. Second, we analyzed district-level aggregated data given its public access. Inevitably, the detailed and nuanced information at the individual level was not considered. Therefore, we suggest that statewide data collection and innovative student assessment systems be utilized to monitor learning progress and identify students’ diverse needs. Third, the study utilized data collected in 2019 and 2021 on state-level standardized tests. However, the pandemic might have a long-term effect on not only students’ academic performance but also their learning motivation and behavior. Future studies should consider collecting longitudinal data to future monitor students’ learning progress and provide students and teachers with timely support.

## Figures and Tables

**Table 1:** STAAR tests criteria across subjects.

Subjects	Test Content	Number of Questions	Total Raw Score	Approaches Grade Level Threshold in 2019	Approaches Grade Level Threshold in 2021
STAAR Grade 5 (G5) Reading Test	Reporting Category 1: Understanding/Analysis Across Genres	8	38	21	21
	Reporting Category 2: Understanding/Analysis of Literacy Texts	16			
	Reporting Category 3: Understanding/Analysis of Informational Texts	14			
STAAR G5 Math Test	Reporting Category 1: Numerical Representations and Relationships	6	36	18	17
	Reporting Category 2: Computation and Algebraic Relationships	17			
	Reporting Category 3: Geometry and Measurement	9			
	Reporting Category 4: Data Analysis and Personal Financial Literacy	4			
STAAR G5 Science Test	Reporting Category 1: Matter and Energy	6	36	22	21
	Reporting Category 2: Force, Motion, and Energy	8			
	Reporting Category 3: Earth and Space	10			
	Reporting Category 4: Organisms and Environments	12			

**Table 2:** Descriptive statistics of STAAR performance by school district location.

		Rural			Non-Rural		
		N	Mean	S.D.	N	Mean	S.D.
%Reading Approach	2019	455	75.69	15.33	691	75.57	11.93
	2021	455	73.63	16.65	690	71.1	13.67
%Math Approach	2019	455	81.28	16.06	691	81.16	12.64
	2021	455	75.21	19.2	690	69.45	I7.26
%Science Approach	2019	454	69.36	19.51	691	71.44	14.45
	2021	455	64.03	19.65	689	61.37	17.86

**Table 3:** *t*-test results from comparing school districts’ demographic characteristics before and after COVID-19.

		Mean	N	S.D.	Sig (2-Tailed)	Cohen’s d
%Instructional Hours	2019	65.44	1152	6.35	0.063	−0.055
	2021	65.58	1152	6.40		
Principal Experience	2019	5.92	1152	3.30	<0.001	−0.064
	2021	6.10	1152	3.32		
Teacher Experience	2019	11.78	1152	3.26	<0.001	−0.133
	2021	12.01	1152	3.10		
T–S Ratio	2019	13.10	1152	2.69	<0.001	0.453
	2021	12.55	1152	2.75		
Teacher Full-Time Equivalence	2019	52.12	1152	6.68	<0.001	0.206
	2021	51.47	1152	6.61		
Teacher Salary	2019	48,459.85	1152	5402.30	<0.001	−1.413
	2021	52,902.63	1152	4837.51		
Turnover Rate	2019	20.84	1149	10.41	<0.001	0.37
	2021	17.23	1149	8.67		
Mobility Rate	2019	14.546	1154	8.32	<0.001	0.355
	2021	12.890	1154	8.51		
%EC	2019	60.482	1154	20.62	0.004	0.086
	2021	59.917	1154	21.05		
%ELs	2019	11.203	1154	13.22	<0.001	−0.314
	2021	11.959	1154	13.80		

**Table 4:** Hierarchical multiple regression analysis predicting students’ learning progress in reading, math, and science from demographic characteristics and locations.

	STAAR_Reading_Approaches Grade Level%				STAAR_Math_Approaches Grade Level%				STAAR_Science_Approaches Grade Level%			
	Model 1		Model 2		Model 1		Model 2		Model 1		Model 2	
Variables	B	β	B	β	B	β	B	β	B	β	B	β
Constant	−4.48		−5.02		−11.76		−12.86		−10.17		−11.27	
Rural	2.41 *	0.09 *	2.04 *	0.08 *	5.77 *	0.18 *	5.07 *	0.15 *	4.82 *	0.14 *	4.42 *	0.13 *
PrExp			0.01	0			0.16	0.03			0.02	0
TExp			0.14	0.02			−0.93 *	−0.09 *			−0.39	−0.04
T–Sratio			−0.1	−0.01			−0.76	−0.06			−1.22 *	−0.09 *
TFulltime			0.01	0			0.23	0.04			0.11	0.02
TSalary			0.0003 *	0.07 *			0.0005 *	0.09 *			0.0003 *	0.07 *
Turnover			0.09 *	0.07 *			0.04	0.02			−0.02	−0.01
Mobility			0.14	0.05			0.13 *	0.04 *			0.32 *	0.09 *
EL			−0.27	−0.04			−0.65	−0.09			−0.53 *	−0.07 *
ED			−0.01	−0.01			0.03	0.01			−0.03	−0.01
*R* ^2^	0.008		0.02		0.031		0.06		0.021		0.044	
*F*	9.69 *		2.50 *		36.046 *		6.535 *		24.296 *		5.223 *	
Δ*R*^2^	0.008		0.013		0.031		0.024		0.021		0.023	
Δ*F*	9.69 *		1.69		36.046 *		3.186 *		24.296 *		3.060 *	

The significance is reported for the following level: * *p* < 0.05.

## Data Availability

The data supporting this study’s outcomes are accessible from the corresponding authors.

## References

[1] Barry D.M., Kanematsu H. (2020). Teaching during the COVID-19 Pandemic. Online Submission. https://files.eric.ed.gov/fulltext/ED606017.pdf.

[2] Haug N., Geyrhofer L., Londei A., Dervic E., Desvars-Larrive A., Loreto V., Pinior B., Thurner S., Klimek P. (2020). Ranking the effectiveness of worldwide COVID-19 government interventions. Nat. Hum. Behav..

[3] Cucinotta D., Vanelli M. (2020). WHO declares COVID-19 a pandemic. Acta Biomed. Atenei Parm..

[4] Kuhfeld M., Soland J., Tarasawa B., Johnson A., Ruzek E., Liu J. (2020). Projecting the potential impact of COVID-19 school closures on academic achievement. Educ. Res..

[5] Hanushek E.A., Woessmann L. (2020). The Economic Impacts of Learning Losses.

[6] Hammerstein S., König C., Dreisörner T., Frey A. (2021). Effects of COVID-19-related school closures on student achievement-a systematic review. Front. Psychol..

[7] Haeck C., Lefebvre P. (2020). Pandemic school closures may increase inequality in test scores. Can. Public Policy.

[8] Garcia E., Weiss E. (2018). COVID-19 and Student Performance, Equity, and US Education Policy: Lessons from Pre-Pandemic Research to Inform Relief, Recovery, and Rebuilding. Economic Policy Institute. https://eric.ed.gov/?id=ED610971.

[9] Adedoyin O.B., Soykan E. (2020). Covid-19 pandemic and online learning: The challenges and opportunities. Interact. Learn. Environ..

[10] Gurung S. (2021). Challenges faced by teachers in online teaching during Covid-19 pandemic. Online J. Distance Educ. E-Learn..

[11] Lemay D.J., Bazelais P., Doleck T. (2021). Transition to online learning during the COVID-19 pandemic. Comput. Hum. Behav. Rep..

[12] Viner R.M., Russell S.J., Croker H., Packer J., Ward J., Stansfield C., Mytton O., Bonell C., Booy R. (2020). School closure and management practices during coronavirus outbreaks including COVID-19: A rapid systematic review. Lancet Child Adolesc. Health.

[13] Swaby A. (2020). Warning of “COVID Slide,” Texas Education Agency Reports 1 in 10 Students have Disengaged during the Pandemic.

[14] Texas Education Agency (2020). Cancellation of STAAR Testing for the Remainder of the School Year. https://tinyurl.com/d4622zok.

[15] Texas Education Agency (2022). Impacts of COVID-19 and Accountability Updates for 2022 and Beyond. https://tea.texas.gov/sites/default/files/2021-tac-accountability-presentation-final.pdf.

[16] Texas Education Agency (2022). Texas Academic Performance Report. https://rptsvr1.tea.texas.gov/perfreport/tapr/tapr_srch.html.

[17] Showalter D., Hartman S.L., Johnson J., Klein B. (2019). Why Rural Matters 2018–2019: The Time Is Now. A Report of the Rural School and Community Trust.

[18] Tang S., Wang Z., Sutton-Jones K.L. (2021). A multilevel study of the impact of district-level characteristics on Texas student growth trajectories on a high-stakes math exam. Mathematics.

[19] Reynolds A.J., Chen C.C., Herbers J.E. School mobility and educational success: A research synthesis and evidence on prevention. Proceedings of the Workshop on the Impact of Mobility and Change on the Lives of Young Children, Schools, and Neighborhoods.

[20] Lowe J.M. (2006). Rural education: Attracting and retaining teachers in small schools. Rural Educ..

[21] Williams S.M., Swain W.A., Graham J.A. (2021). Race, climate, and turnover: An examination of the teacher labor market in rural Georgia. AERA Open.

[22] Glover T.A., Nugent G.C., Chumney F.L., Ihlo T., Shapiro E.S., Guard K., Koziol N., Bovaird J. (2016). Investigating Rural Teachers’ Professional Development, Instructional Knowledge, and Classroom Practice. J. Res. Rural Educ..

[23] Vernon-Feagans L., Bratsch-Hines M., Varghese C., Cutrer E.A., Garwood J.D. (2018). Improving struggling readers’ early literacy skills through a Tier 2 professional development program for rural classroom teachers: The targeted reading intervention. Elem. Sch. J..

[24] Showalter D., Klein R., Johnson J., Hartman S.L. (2017). Why Rural Matters 2015–2016: Understanding the Changing Landscape. A Report of the Rural School and Community Trust.

[25] Cantrell S.C., Rintamaa M., Anderman E.M., Anderman L.H. (2018). Rural adolescents’ reading motivation, achievement and behavior across transition to high school. J. Educ. Res..

[26] Holland G., Mann M., Daniels C., Hester J. (2011). Elementary Science Student Achievement and School District Enrollment Sizes in Texas. Schooling.

[27] Korkmaz S., Kazgan A., Çekiç S., Tartar A.S., Balcı H.N., Atmaca M. (2020). The anxiety levels, quality of sleep and life and problem-solving skills in healthcare workers employed in COVID-19 services. J. Clin. Neurosci..

[28] Prince O.M. (2021). Addressing teaching and learning challenges amidst the Covid 19 pandemic: Implications for the Nigeria Primary Schools. Int. J. Integr. Educ..

[29] Darling-Hammond L., Hyler M.E. (2020). Preparing educators for the time of COVID and beyond. Eur. J. Teach. Educ..

[30] Mishra L., Gupta T., Shree A. (2020). Online teaching-learning in higher education during lockdown period of COVID-19 pandemic. Int. J. Educ. Res. Open.

[31] McMahon D.E., Peters G.A., Ivers L.C., Freeman E.E. (2020). Global resource shortages during COVID-19: Bad news for low-income countries. PLoS Neglected Trop. Dis..

[32] Supovitz J.A., Hemphill A.A., Manghani O., Watson C. (2023). Cogs of inequity: How structural inequities impeded school efforts to support students and families at the outbreak of the COVID-19 pandemic. Equity Educ. Soc..

[33] Flaxman S., Mishra S., Gandy A., Unwin HJ T., Mellan T.A., Coupland H., Vollmer MA C. (2020). Estimating the effects of non-pharmaceutical interventions on COVID-19 in Europe. Nature.

[34] McIntosh C.E., Brelage P.K., Thomas C.M., Wendel J.M., Phelps B. (2022). School nurse and COVID-19 response. Psychol. Sch..

[35] Dadhe P.P., Kuthe G.D. (2021). Assessment of Availability and Use of Technology by Students for Online Education during COVID-19 Pandemic in Rural India: A Case Study. Libr. Philos. Pract..

[36] Bacher-Hicks A., Goodman J., Mulhern C. (2021). Inequality in household adaptation to schooling shocks: Covid-induced online learning engagement in real time. Econ. Educ. Rev..

[37] Dombo E.A., Sosa L.V., Sabatino C. (2021). When a Crisis Becomes the New Normal: Supporting Children, Families, and Schools during and after COVID-19. Child. Sch..

[38] Francis D.V., Weller C.E. (2021). Economic Inequality, the Digital Divide, and Remote Learning During COVID-19. Rev. Black Political Econ..

[39] Korkmaz G., Toraman Ç. (2020). Are we ready for the post-COVID-19 educational practice? An investigation into what educators think as to online learning. Int. J. Technol. Educ. Sci..

[40] Supovitz J.A., Manghani O. (2022). The Role of Inequity in School Responses to the COVID-19 Pandemic. Peabody J. Educ..

[41] Anderson L.W. (2021). Schooling interrupted: Educating children and youth in the COVID-19 Era. CEPS J..

[42] D’Amico M.M., Atwell A.K., Spriggs J.N., Cox J.A. (2022). Are we doing enough? COVID responses from urban and rural community colleges. Community Coll. J. Res. Pract..

[43] Ruiz R., Perna L.W. (2017). The geography of college attainment: Dismantling rural “disadvantage”. Indic. High. Educ. Equity United States.

[44] Gagnon D.J., Mattingly M.J. (2018). Racial/Ethnic test score gaps and the urban continuum. J. Res. Rural Educ..

[45] Wang Z., Tang S., Sutton-Jones K. (2019). Texas rural vs. nonrural school district student growth trajectories on a high-stakes science exam: A multilevel approach. Soc. Sci..

[46] Tang S., Wang Z., Sutton-Jones K.L. (2023). A multi-level analysis of upper elementary students’ performance on the STAAR reading exam: Comparing growth trajectories of rural and non-rural school districts. Educ. Stud..

[47] Mercier K., Centeio E., Garn A., Erwin H., Marttinen R., Foley J. (2021). Physical education teachers’ experiences with remote instruction during the initial phase of the COVID-19 pandemic. J. Teach. Phys. Educ..

[48] Kaden U. (2020). COVID-19 school closure-related changes to the professional life of a K–12 teacher. Educ. Sci..

[49] Poletti M., Raballo A. (2021). Coronavirus disease 2019 and effects of school closure for children and their families. JAMA Pediatr..

[50] Drescher J., Podolsky A., Reardon S.F., Torrance G. (2022). The Geography of Rural Educational Opportunity. RSF Russell Sage Found. J. Soc. Sci..

[51] Johnson A., Kuhfeld M., Soland J. (2021). The Forgotten 20%: Achievement and Growth in Rural Schools Across the Nation. AERA Open.

[52] Greenough R., Nelson S.R. (2015). Recognizing the variety of rural schools. Peabody J. Educ..

[53] Strange M. (2011). Finding fairness for rural students. Phi Delta Kappan.

[54] Texas Education Agency (2024). STAAR Performance Standards. https://tea.texas.gov/student-assessment/testing/student-assessment-results/staar-performance-standards#:~:text=in%20familiar%20contexts.-,Approaches%20Grade%20Level,and%20skills%20in%20familiar%20contexts.

[55] Ronfeldt M., Loeb S., Wyckoff J. (2013). How teacher turnover harms student achievement. Am. Educ. Res. J..

[56] Titus D.N. (2007). Strategies and resources for enhancing the achievement of mobile students. Nassp Bull..

[57] Chen Y., Cao L. (2022). Promoting maker-centred instruction through virtual professional development activities for K-12 teachers in low-income rural areas. Br. J. Educ. Technol..

[58] Locke V.N., Patarapichayatham C., Lewis S. (2021). Learning Loss in Reading and Math in US Schools Due to the COVID-19 Pandemic.

[59] García E., Han E.S. (2022). Teachers’ Base Salary and Districts’ Academic Performance: Evidence From National Data. SAGE Open.

[60] Korman H.T., O’Keefe B., Repka M. (2020). Missing in the Margins: Estimating the Scale of the COVID-19 Attendance Crisis. Bellwether Education Partners. https://bellwethereducation.org/.

[61] Chetty R., Friedman J.N., Hendren N., Stepner M. (2020). Real-Time Economics: A New Platform to Track the Impacts of COVID-19 on People, Businesses, and Communities Using Private Sector Data. NBER Working Paper 27431. https://www.nber.org/.

